# Effect of Age on Bone Structure Parameters in Laying Hens

**DOI:** 10.3390/ani11020570

**Published:** 2021-02-22

**Authors:** Masayoshi Yamada, Chongxiao Chen, Toshie Sugiyama, Woo Kyun Kim

**Affiliations:** 1 Graduate School of Science and Technology, Niigata University, 2-8050 Ikarashi, Nishi-ku, Niigata 950-2181, Japan; f19d041a@gmail.com (M.Y.); sugiyama@agr.niigata-u.ac.jp (T.S.); 2 Prestage Department of Poultry Science, North Carolina State University, Raleigh, NC 27695, USA; sean_chen@ncsu.edu; 3 Department of Poultry Science, University of Georgia, Athens, GA 30606, USA

**Keywords:** laying hens, osteoporosis, medullary bone, aging

## Abstract

**Simple Summary:**

Laying hens supply calcium to their eggshells via a process of dynamic bone formation and resorption called remodeling. As hens age, their bone becomes weak and brittle due to an imbalance in remodeling, resulting in bone disorders. Osteoporosis, characterized by bone mass loss and increased risk of fractures, is a serious problem in the egg industry, affecting both productivity and animal welfare. Nonetheless, the mechanisms of age-related osteoporosis remain unknown. We evaluated differences in bone structure between age groups, using microscopy and 3D imaging. Bone quality was reduced by an increase in porosity, with loss of volume, contributing to age-related osteoporosis.

**Abstract:**

Changes in medullary and cortical bone structure with age remain unclear. Twenty Hy-Line W36 hens, 25 or 52 weeks of age, were euthanized, and both tibiae were collected when an egg was present in the magnum. Serial cross sections of the tibiae were stained with Alcian blue. The bones were scanned using micro-computed tomography. Trabecular width (Tb.Wi) was significantly higher (*p* < 0.05) in 25-week-old hens, whereas medullary bone tissue volume (TV) was significantly higher (*p* < 0.01) in 52-week-old hens. 25-week-old hens had significantly higher (*p* < 0.01) bone volume fraction (BVF = calcified tissue / TV). Moreover, the cortical bone parameters were significantly higher (TV and bone mineral content (BMC) at *p* < 0.05, and bone volume (BV) and BVF at *p* < 0.01) in younger hens. Open porosity and total porosity, which indicate less density, were significantly higher (*p* < 0.01) in older hens. Older hens showed significantly higher (*p* < 0.01) tibial diaphysis TV than younger hens. Younger hens had significantly higher (*p* < 0.01) BV, BVF and bone mineral density (BMD) of the tibial diaphysis. These findings reveal that reductions in medullary bone quality might be associated with age-related low estrogen levels and stimulation of osteoclastic bone resorption by parathyroid hormone. Cortical bone quality decreased with enlargement of the Haversian canals and loss of volume, with a longer egg-laying period leading to osteoporosis.

## 1. Introduction

Laying hens have a unique mechanism of eggshell calcification. Medullary bone is a woven bone that plays a critical role as a calcium reservoir for eggshell [1,2,3]. However, it is mechanically weaker than structural (cortical and cancellous) bone, without contributing to bone strength [4,5]. This bone is rapidly remodeled during the egg-laying cycle; it is actively formed in the bone marrow before the onset of eggshell calcification in the shell gland [2]. Estrogen is crucial for medullary bone development. Endosteal bone-lining cells express estrogen receptors; estrogen affects their differentiation into osteoblasts [6,7]. Parathyroid hormone (PTH) is upregulated in response to low blood calcium resulting from high calcium demand on eggshell calcification [8]. PTH stimulates osteoclastic bone resorption in the medullary bone, increasing blood calcium level [9]. Osteoclasts perform bone resorption by decalcifying the inorganic matrix and digesting the organic matrix of the medullary bone [10,11].

Laying hens lose their structural bone during the late laying period [12], leading to serious skeletal problems such as osteoporosis, affecting animal health and welfare and causing economic consequences [13,14]. Osteoporosis refers to structural bone loss that increases the risk of fractures [4]. During the laying period, enhance medullary bone volume and loss structural bone volume is occurred [3].

Micro-computed tomography (micro-CT) scanning, a 3D-imaging technique for bone structural analysis, is used to detect osteoporosis. Cortical and medullary bone morphology can be measured separately, allowing the researcher to select a smaller region of interest [12]. Bone mineral density (BMD) and bone mineral content (BMC) increase linearly with age [14]. Structural bone quality cannot be properly assessed without also considering medullary bone, even though it does not affect bone strength.

This study aimed to determine age-related differences in medullary bone quality during ossification, by examine bone histology and morphology. The second aim was to profile cortical bone quality in hens during extended laying periods.

## 2. Materials and Methods

### 2.1. Animals, Hosusing, and Diet

A total of 20 Hy-Line W36 hens at 18 weeks of age were used for this study. The diets were made according to Hy-Line W36 guide (2015) and are shown in Table 1. Water and diet were freely accessed. All hens were kept in individual pens (41 × 26 × 46 cm), providing artificial light with 15.5 h and dark with 8.5 h in closed houses at the research facility of the Department of Poultry Science at the University of Georgia. The onset of egg-laying was at 18-week-old. All hens were euthanized at 25 or 52 weeks of age (10 birds each) by cervical dislocation when eggs were in their magnums (3 to 4 h after oviposition). For consistency, an egg-laying record was daily kept on all hens to confirm their egg-laying cycle for at least 4 weeks before sacrifice. If any irregularities were noted, the hens were not used for this experiment. All hens used here were sacrificed at 3 to 4 h after oviposition of the first egg in a clutch. Concomitantly, the presence of an egg in magnum was ascertained by autopsy. Both tibiae were collected and immediately fixed at 10% buffer formalin. The portion of epiphyses were removed and diaphyses were used for histological and micro-CT analysis after fixation. The study was approved by Institutional Animal Care and Use Committee at the University of Georgia (A2016 11-003).

### 2.2. Histological Analysis

Left tibial diaphyses were cut into seven sections and each fourth section was used for histological analysis. These were decalcified using Plank-Rychlo’s solution, dehydrated, and embedded in paraffin wax. Serial cross-sections of 5 μm thick were obtained for Alcian blue staining, which detects medullary bone [15]. The slides were visualized at 40× magnification using a microscope equipped with a Leica DC 500 camera (Leica Microsystems, Wetzlar, Germany). Histomorphometry was performed for five randomly selected non-overlapping microscopic fields, using ImageJ software, version 1.46r (National Institutes of Health, Bethesda, MD, USA). Total area (T.Ar), medullary bone area (B.Ar), and medullary bone perimeter (B.Pm) were measured. B.Ar/T.Ar, the ratio of medullary bone area to total bone marrow area, is expressed as a percentage. Trabecular number (Tb.N), width (Tb.Wi), and spacing (Tb.S) were calculated as previously described [16]. Five readings in each section were averaged for data analysis.

### 2.3. Micro-Computed Tomography Analysis

Right tibial diaphyses were rapidly washed in PBS, and were scanned using Sky scanner 1275 (Bruker, Billerica, MA, USA) at 75 kV and 133 μA. The 2D images were reconstructed using NRecon Reconstruction-software, version 1.6 (Bruker, USA). A volume-of-interest (VOI) was selected up and down from the center of each diaphysis in total of 200 slides (5 mm), and 3D image analysis was performed using CTAN, version 1.10 (Bruker, USA). Tissue volume (TV), bone volume (BV), bone volume fraction (BVF = BV/TV), bone mineral content (BMC), bone mineral density (BMD) of the tibial diaphyses were measured.

The VOI contains both cortical and medullary bone, and these were separately analyzed [12]. TV, BV, BVF, BMC and BMD of cortical bone were measured. Open porosity (%), close porosity (%) and total porosity (%), describing the volume of pores in the cortical bone compartments, were also measured. In the medullary bone compartment, TV, BV, BVF, BMC, BMD of medullary bone were measured.

### 2.4. Statistical Analysis

Data represent the mean ± standard error (SE). All data were analyzed using Stu-dent’s *t*-tests in JMP, version 9.0.0 (SAS Institute, Greenwood Village, CO, USA).

## 3. Results

### 3.1. Histological Analysis

**Figure 1:**
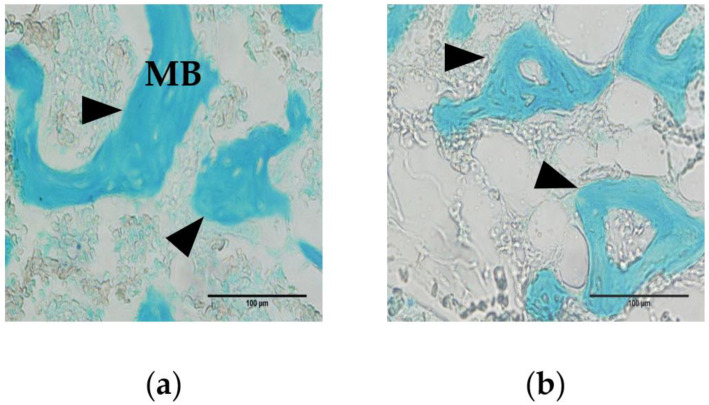
Alcian blue staining of tibial sections from Hy-Line W36 hens. Medullary bone (MB) was observed in the bone marrow cavity. (**a**) In 25-week-old hens, thickened trabeculae were observed (arrowhead); (**b**) In 52-week-old hens, thinner trabeculae were observed.

The medullary bone was reticulately developed in the bone marrow cavity (Figure 1, blue-stained area). The trabeculae were thicker and better developed in 25-week-old hens (Figure 1a) than in 52-week-old hens (Figure 1b). There were no significant differences in B.Ar/T.Ar, B.Ar, B.Pm, Tb.N, or Tb.S between the two age groups (Table 2). Tb.Wi was significantly higher (*p* < 0.05) in 25-week-old hens than in 52-week-old hens.

### 3.2. Micro-Computed Tomography Analysis

Medullary bone TV was significantly higher (*p* < 0.01) in 52-week-old than in 25-week-old hens, whereas BVF was significantly higher (*p* < 0.01) in 25-week-old than in 52-week-old hens, indicating that older hens had bigger marrows but less bones in a unit of marrow volume. There were no significant differences in BV, BMD or BMC between the two age groups (Table 3).

25-week-old hens had significantly higher cortical bone TV and BMC (*p* < 0.05), and BV and BVF (*p* < 0.01), than 52-week-old hens (Table 4). Cortical bone open porosity and total porosity, which indicate less density, were significantly higher (*p* < 0.01) in 52-week-old than in 25-week-old hens. There were no significant differences in BMD or Close porosity between the two age groups.

Tibial diaphysis TV was significantly higher (*p* < 0.01) in 52-week-old than in 25-week-old hens, whereas 25-week-old hens had significantly higher BV, BVF, BMD (*p* < 0.01) than 52-week-old hens, indicating that aging increases bone size but decreases bone mineralization. There were no significant differences in BMC between the two age groups (Table 5).

## 4. Discussion

Medullary bone development was evaluated using histological and morphological examination, to compare ossification in the two age groups. Trabecular thickness is obtained from trabecular width (Tb.Wi) via a stereological formula [16]. Tb.Wi and bone volume fraction (BVF) of medullary bone decreased with age (Table 2 and Table 3). Estrogen administration increases medullary bone mass in a concentration-dependent manner in a female avian model [6]. Aging reduces estrogen synthesis by the ovaries, and aged hens have low serum estrogen levels [17]. Medullary bone volume decreases with age [18]. Thus, age-dependent low estrogen levels could inhibit medullary bone formation, potentially limiting the calcium supply for eggshells. Moreover, estrogen prevented the loss of cancellous bone thickness in an ovariectomized mouse model [19]. Therefore, changes in estrogen levels might be responsible for medullary bone trabecular development [6]. On the other hand, estrogen is closely correlated with parathyroid hormone (PTH), a principal hormone of osteoclastic bone resorptive stimulator [20,21]. Administration of PTH stimulates development of osteoclast ruffled border which secretes enzyme and acid for bone resorption both in vitro and in vivo [22,23]. Feeding calcium deficient diet to laying hens, the increased serum PTH and accumulation of poorly calcified medullary bone were observed with low serum calcium and estrogen levels [24]. Two age groups were fed different levels of calcium containing 4.94% Ca for 25-week-old and 4.40% Ca for 52-week-old. In addition, it is reported that intestinal and renal calcium absorption capacity declined with age [25]. Therefore, the volume and trabecular width of calcified medullary bone were decrease by hormonal changes related with calcium metabolism.

For the cortical portion of the tibia, tissue volume (TV) and bone volume (BV) were significantly lower in the older hens (Table 4), suggesting that cortical bone resorption occurs due to the higher calcium demands of producing eggs rather than medullary bone. As a result, prolonged egg production damages skeletal integrity and contributes to serious structural bone loss, because structural bone is not restored during the laying period [3,26,27]. The older birds had lower bone volume fraction (BVF) and mineral content (BMC), significantly higher open porosity and total porosity, and tended to have higher closed porosity (Table 4), possibly due to enlargement of the Haversian canals in the cortical bone. The Haversian canals are vertical channels in cortical bone for blood vessels and are surrounded by bone matrix and osteocytes [28]. In cortical bone remodeling, osteoclasts resorb bone to widen the Haversian canal; osteoblasts form layers of bone, becoming buried in the bone matrix as osteocytes [29]. Feeding calcium deficient diet to laying hens, the amount of medullary bone ash remains constant, that of cortical bone decreased on the other hand [30]. In addition, low calcium diet increase serum parathyroid hormone (PTH) level and decrease that of estrogen in laying hens [24]. Estrogen affects osteoclasts via their estrogen receptors, reversing bone absorption [6]; therefore, estrogen deficiency might activate osteoclastic resorption by PTH, resulting in enlargement of the cortical bone Haversian canals of the cortical bone. Osteoclastic resorption in aged hens results in enlarged Haversian canals with a perforated structure (porosity) [31]. Further, the number of osteoclasts in medullary bone decreases with age in hens [32]. These facts explain why the cortical bone becomes thinner and more porous with an extended egg-laying period.

We observed that tibial diaphysis bone volume (BV), the bone volume fraction (BVF), and bone mineral density (BMD) were lower in the older group (Table 5). It is reported BMD has a correlation with increase in accumulation of calcified medullary bone [33]. Trabecular width (Tb.Wi) and bone volume fraction (BVF) of the medullary bone decreased in the older group. Low dietary calcium associates with less amount of medullary bone, decreasing in total BMD [34]. Therefore, medullary bone loss by hormonal changes related calcium metabolism deteriorated BMD of tibial diaphysis. In addition, loss of cortical bone volume (BV) and BVF might decrease that of tibial diaphysis. However, BVF and BMD of whole bone, including the tibial epiphysis, which is rich in cancellous bone increased linearly with age in previous research [18,35]. It is suggested that these results might depend on the part of bone to be analyzed. This study provides a detailed analysis of the tibial diaphysis in hens, focusing separately on medullary and cortical bone, via micro-CT scanning [12].

## 5. Conclusions

Medullary bone quality was lower in the older group of White Leghorn hens, possibly due to osteoblast dysfunction resulting from a low estrogen levels and stimulation of osteoclastic bone resorption by parathyroid hormone (PTH). Cortical bone quality is lower during an extended egg-laying period. These factors caused the older hens to have poorer bone quality, with enlarged Haversian canals and lower bone volume.

These findings reveal that reductions in medullary bone quality might be associated with age-related low estrogen levels and stimulation of osteoclastic bone resorption by parathyroid hormone. Cortical bone quality decreased with enlargement of the Haversian canals and loss of volume, with a longer egg-laying period leading to osteoporosis.

## Figures and Tables

**Table 1:** Composition of diet from 18 to 52 weeks of age.

Ingredients, %	^1^ Peaking	^2^ Layer1	^3^ Layer2
Corn	53.61	62.99	61.54
Soybean meal—48%	28.10	21.35	19.99
Soybean oil	3.75	2.90	3.00
Limestone	7.44	6.89	6.87
Oyster shell	3.19	2.95	2.94
Defluorinated phosphate	2.55	2.09	1.89
Common salt	0.30	0.30	0.30
L-Lyshine HCL	0.46	0.09	0.04
DL-Methionine	0.33	0.22	0.17
Threonine	0.11	0.06	0.03
Vitamin D3 (IU/kg diet)	2760	2760	2760
^4^ *Vitamin premix*	0.05	0.05	0.05
^5^ *Mineral premix*	0.06.	0.06	0.06
Sand	0.05	0.05	3.11
ME (kcal/kg diet)	2840	2900	2820
CP %	19.05	16.15	15.27
Ca %	4.94	4.48	4.40
Available P (%)	0.58	0.49	0.45

^1^ Peaking: hens from 18 to 38-weeks of age, ^2^ Layer1: hens from 39 to 48 weeks of age, ^3^ Layer2: hens from 49 to 52 weeks of age, ^4^ Vitamin premix: supplied per kilogram of diet (vitamin A, 9900 IU; vitamin E, 22.10 IU; vitamin B12, 0.02 mg; biotin, 0.06 mg; menadione, 3.3 mg; thiamine, 2.20 mg; riboflavin, 6.60 mg; pantothenic acid, 11.00 mg; vitamin B6, 4.40 mg; niacin, 33.00 mg; folic acid, 0.90 mg; choline, 191.36 mg), ^5^ Mineral premix: supplied per kilogram of diet (Mn, 80.4 mg; Zn, 64.2 mg; Mg, 16.08 mg; Fe, 15.78 mg; Cu, 2.4 mg; I, 0.6 mg; Se, 0.24 mg).

**Table 2:** Histological parameters of Hy-Line W36 hens tibial cross-sections.

Measurement	25-wk	52-wk	*p*-Value
^1^ B.Ar/T.Ar (%)	25.5 ± 1.22	28.3 ± 0.834	0.073
^2^ B.Ar (μm²)	8585 ± 550	9301 ± 320	0.280
^3^ B.Pm (μm)	980 ± 63.3	1060 ± 36.0	0.265
^4^ Tb.Wi (μm)	0.01752 ± 0.011	0.01748 ± 0.007	0.016
^5^ Tb.N (/N)	13.2 ± 0.856	14.4 ± 0.486	0.265
^6^ Tb.Sp (μm)	68.5 ± 9.47	62.6 ± 5.32	0.594

^1^ B.Ar/T.Ar: medullary bone area as a proportion of the total cross-sectional area; ^2^ B.Ar: medullary bone area; ^3^ B.Pm: medullary bone perimeter; ^4^ Tb.Wi: trabecular width: ^5^ Tb.N: trabecular number; ^6^ Tb.Sp.: trabecular spacing. *p* < 0.05, comparing the age groups. Results were presented as mean ± SE.

**Table 3:** Morphology, based on micro-computed tomography (micro-CT) scanning, of medullary bone from Hy-Line W36 hens.

Measurement	25-wk	52-wk	*p*-Value
^1^ TV (cm³)	211 ± 10.2	285 ± 11.1	0.0002
^2^ BV (cm³)	73.7 ± 10.5	38.6 ± 13.6	0.064
^3^ BVF (%)	35.2 ± 4.54	13.9 ± 4.84	0.006
^4^ BMD (g/cm³)	0.160 ± 0.014	0.147 ± 0.014	0.510
^5^ BMC (g)	33.7 ± 3.35	41.0 ± 3.27	0.074

^1^ TV: tissue volume; ^2^ BV: bone volume; ^3^ BVF: bone volume fraction (BV/TV); ^4^ BMC: bone mineral content; ^5^ BMD: bone mineral density. *p* < 0.01, comparing the age groups. Results were presented as mean ± SE.

**Table 4:** Morphology, based on micro-CT scanning, of cortical bone from Hy-Line W36 hens.

Measurement	25-wk	52-wk	*p*-Value
^1^ TV (cm³)	172 ± 7.04	145 ± 8.57	0.028
^2^ BV (cm³)	172 ± 7.00	140 ± 7.43	0.007
^3^ BVF (%)	99.7 ± 0.078	96.7 ± 0.967	0.009
^4^ BMD (g/cm³)	1.26 ± 0.009	1.24 ± 0.014	0.098
^5^ BMC (g)	217 ± 10.0	180 ± 9.69	0.017
Closed Porosity (%)	0.0145 ± 0.005	0.117 ± 0.058	0.119
Open porosity (%)	0.255 ± 0.078	3.22 ± 0.930	0.009
Total porosity (%)	0.269 ± 0.078	3.33 ± 0.967	0.009

^1^ TV: tissue volume; ^2^ BV: bone volume; ^3^ BVF: bone volume fraction (BV/TV); ^4^ BMC: bone mineral content; ^5^ BMD: bone mineral density. *p* < 0.05, *p* < 0.01, comparing the age groups. Results were presented as mean ± SE.

**Table 5:** Morphology, based on micro-CT scanning, of tibial diaphysis from Hy-Line W36 hens.

Measurement	25-wk	52-wk	*p*-Value
^1^ TV (cm³)	383 ± 11.2	430 ± 10.7	0.008
^2^ BV (cm³)	246 ± 13.9	178 ± 15.7	0.006
^3^ BVF (%)	64.1 ± 3.13	41.6 ± 3.83	0.0004
^4^ BMD (g/cm³)	0.655 ± 0.0240	0.514 ± 0.0272	0.002
^5^ BMC (g)	250 ± 10.6	221 ± 11.5	0.074

^1^ TV: tissue volume ^2^ BV; bone volume; ^3^ BVF: bone volume fraction (BV/TV); ^4^ BMC: bone mineral content, ^5^ BMD: bone mineral density. *p* < 0.01, comparing the age groups Results were presented as mean ± SE.

## Data Availability

The data presented in this study are available on request from the corresponding author.
